# How to identify respiratory pathogens in primary health care - a review on the benefits, prospects and pitfalls in using point of care tests

**DOI:** 10.1007/s15010-025-02600-1

**Published:** 2025-07-09

**Authors:** Manfred Nairz, Guenter Weiss

**Affiliations:** Department of Internal Medicine II (Infectious Diseases, Immunology, Rheumatology, Pneumology), Medical University of Innsbruck, Anichstr. 35, Innsbruck, A-6020 Austria

**Keywords:** Antimicrobial stewardship, Community-acquired pneumonia, Diagnostic stewardship, Polymerase chain reaction, Rapid antigen detection test, Respiratory tract infection

## Abstract

**Purpose:**

Respiratory tract infections are among the most common reasons for consultations in primary health care (PHC) settings. In this review, we aim to provide an overview of diagnostic tests for selected respiratory pathogens useful in PHC.

**Methods:**

We performed a PubMed search on diagnostic tests for influenza virus, respiratory syncytial virus (RSV), Severe Acute Respiratory Syndrome Coronavirus type 2 (SARS-CoV-2), *Streptococcus pneumoniae*, *Legionella pneumophila*, *Mycoplasma pneumoniae* and *Bordetella pertussis*. We then selected and summarized clinical trials, meta-analyses and systematic reviews published between May 1994 and April 2025 relevant to PHC.

**Results:**

Diagnostic tests are useful if the test result will guide subsequent clinical management. Polymerase chain reaction (PCR) tests have high diagnostic accuracy but are not always available in PHC. Accurate rapid antigen detections tests (RADTs) are required to have a sensitivity of at least 80% and a specificity of at least 97% and are available for influenza virus, RSV and SARS-CoV-2 as are urinary antigen tests for *Streptococcus pneumoniae* and *Legionella pneumophila*. In contrast, due to the lack of appropriate RADTs, infections with *Mycoplasma pneumoniae* or *Bordetella pertussis* typically require PCR tests.

**Conclusion:**

From a clinical perspective, the differentiation between viral and bacterial infections and the accurate identification of the specific causative agent may guide medical interventions including antimicrobial therapy. From a diagnostic perspective, adequate microbiologic sampling and careful interpretation of laboratory test results in a clinical context are central requirements.

## Introduction

Respiratory tract infections (RTIs) are among the leading causes of morbidity worldwide [1]. They encompass a broad spectrum of clinical presentations, ranging from mild viral illnesses such as the common cold to potentially life-threatening conditions like community-acquired pneumonia (CAP) [2, 3]. In Western countries, symptoms and signs of RTIs such as cough and fever are a frequent cause why patients seek medical attention in PHC settings, accounting for 5 to 25% of all visits [4–7].

Approximately two thirds of RTIs in PHC patients are caused by respiratory viruses such as influenza viruses, RSV, SARS-CoV-2, other human coronaviruses, human rhinovirus or human metapneumovirus [8–12]. Bacterial aetiologies of RTIs include *Streptococcus* (*S*.) *pneumoniae*, *Haemophilus* (*H*.) *influenzae*, *Mycoplasma* (*M*.) *pneumoniae*, *Chlamydia* (*C*.) *pneumophila*, *Moraxella catarrhalis*, *Bordetella* (*B*.) *pertussis* and *Legionella* (*L*.) *pneumophila* [13, 14]. Furthermore, co-infections with more than one viral or bacterial pathogen are increasingly detected [15, 16], especially with molecular testing using multiplex PCR or next generation sequencing [17, 18].

Multiplex PCR uses multiple primers to detect several organisms in a single assay. From a practical standpoint, multiplex assays can be grouped into point of care (POC)-type assays for influenza virus/RSV/SARS-CoV-2 and into more comprehensive syndromic panels for upper RTIs (covering many respiratory viruses along with atypical pathogens *M*. *pneumoniae*, *C. pneumophila* and *B. pertussis*) or lower RTIs (covering causative agents for pneumonia such as *S*. *pneumoniae*, *H*. *influenzae*, *Klebsiella* species, *Proteus* species, *Escherichia coli*, *Pseudomonas aeruginosa*, *Acinetobacter baumannii* complex, *Serratia marcescens* and *Staphylococcus aureus*, respectively [19, 20]. The latter group of tests is mainly relevant to hospital settings and is thus not within the scope of this review [21, 22].

PHC provides the first contact with the health care system when a health problem occurs. PHC thus compasses a variety of settings and providers including general practitioner (GP) offices, PHC centers, emergency rooms and community pharmacies each of which may face different logistic challenges and diagnostic needs. The use of point of care tests (POCT) systems varies considerably between Western countries [23]. This may be attributable to differences in the adoption of test systems, the assessment of their clinical utility and risk as well as in reimbursement between countries [24]. Furthermore, the staffing and staff training level may be different between countries [25, 26].

Up to 90% of RTI cases are managed in PHC [27]. An accurate and timely diagnosis of RTIs is considered to be key in modern medicine, especially in severely ill or immunocompromised patients or during outbreaks with specific pathogens [28]. The identification of causative respiratory pathogens is important for treatment decisions, guides the management of patient flows in PHC centers and other healthcare facilities, and for infection control measures to prevent further transmissions [29]. Clinicians in PHC settings often encounter substantial diagnostic uncertainty when evaluating patients with RTIs [30]. Clinical signs and symptoms alone may be nonspecific, making it difficult to distinguish between bacterial and viral infections or to identify specific causative agents [14, 31]. Financial or logistic reasons may limit the access to diagnostic technologies in PHC [32], and physicians face time constraints and the pressure to make quick treatment decisions [33].

The rational indication, patient selection and use of diagnostic tests by trained healthcare professionals minimizes errors and facilitates the accurate interpretation of test results for the diagnosis of specific RTIs [34–37]. Moreover, the appropriate choice and efficient use of diagnostic tests can prevent unnecessary treatments and hospitalizations, thus reducing the overall burden on healthcare systems [38].

In this review, we summarize clinical trials, meta-analyses and systematic reviews on selected diagnostic tests to be used at the point of care for clinically relevant respiratory pathogens. Many RADTs for Influenza virus, RSV and SARS-CoV-2 are accurate and suitable for PHC settings. In addition, there are urinary antigen tests (UATs) for *S. pneumoniae* and *L. pneumophila*. In contrast, we largely lack simple test systems for *M. pneumoniae* and *B. pertussis* that are easy to use at the point of care. In this review, we will focus on the principles, advantages, limitations, and clinical applications of RADTs for use in PHC centers. We also indicate where samples for PCR or serologic tests should be sent to an infectious disease (ID) or microbiologic laboratory for further diagnostic work-up.

## Main

In recent years, there have been considerable advances in diagnostic testing including nucleic acid amplification techniques such as PCR and loop-mediated isothermal amplification (LAMP). As a consequence, various POCT systems now offer the potential for rapid, near-patient diagnosis of specific RTIs in PHC [11, 39–42].

Given the growing complexity of diagnostic decision-making in PHC and the critical importance of accurate pathogen detection, there is a need to summarize the benefits, prospects and pitfalls of current diagnostic approaches for RTIs in PHC settings. We therefore performed a search in PubMed on clinical trials, meta-analyses and systematic reviews of diagnostic tests for influenza virus, respiratory syncytial virus (RSV), Severe Acute Respiratory Syndrome Coronavirus type 2 (SARS-CoV-2), *S. pneumoniae*, *L. pneumophila*, *M. pneumoniae* and *B. pertussis*. We then selected those published between May 1994 and April 2025 which are relevant to PHC.

### When is the diagnostic accuracy of rapid antigen detection tests sufficient?

As immunological tests, RADTs have generally lower analytical sensitivity compared to molecular tests such as PCR (Table 1) and LAMP [43]. This lower sensitivity can potentially lead to false-negative test results, especially in asymptomatic individuals, in early stages of disease, in conditions of low pathogen load and in samples of poor quality [44–46]. This being said, it remains uncertain whether or not RADTs should be used to screen asymptomatic individuals such as subjects with close contact to patients with respiratory infections [47]. Furthermore, a negative RADT– even in the setting of high pre-test probability– does not rule out infection and needs eventual confirmation by PCR [48]. However, most RADTs have sufficiently high specificity which is crucial for reducing false-positive rates, thus avoiding unnecessary treatment or isolation.

**Table 1:** Selected advantages and disadvantages of diagnostic POCT

Test principle	Pros	Cons	Comments
Antigenic tests	Rapid results, no major equipment needed, easy to use, cheap [49, 52].	Sensitivity varies widely by manufacturer and product, sensitivity may change with serotype or virus variants [97, 150].	Only to be used in symptomatic patients, may be negative in early stages of the disease, systems using fluorescence and an automated reader may provide higher sensitivity [46, 50, 51].
PCR	Today’s gold standard for the detection of several respiratory pathogens [54, 77, 139, 154, 233].	Technical equipment needed.	Moderate to high costs. Not always reimbursed.
Serologic tests	Available for many respiratory pathogens enabling parallel or sequential work-up.	Diagnostic gap due to sero-conversion, typically requires two serum samples taken 2 weeks apart [234, 235].	Limited use for POCT in PHC settings due to intermediate turnaround times, preferable for epidemiological studies and for post-hoc diagnosis.
Multiplex molecular tests	Can be considered in critically-ill patients [88, 89].	May yield test results without therapeutic consequences [236, 237].	Codetections hamper the identification of the leading cause of disease, resistance markers need to be correctly associated with a specific pathogen [238].

RADTs offer rapid results, often within 10 to 20 min. This facilitates immediate clinical decision-making in PHC centers [49]. Negative RADT results have a good predictive value in ruling out transmission-relevant infections, however, sensitivity and specificity may greatly vary among test systems from different manufacturers [46, 50, 51].

**Fig. 1:**
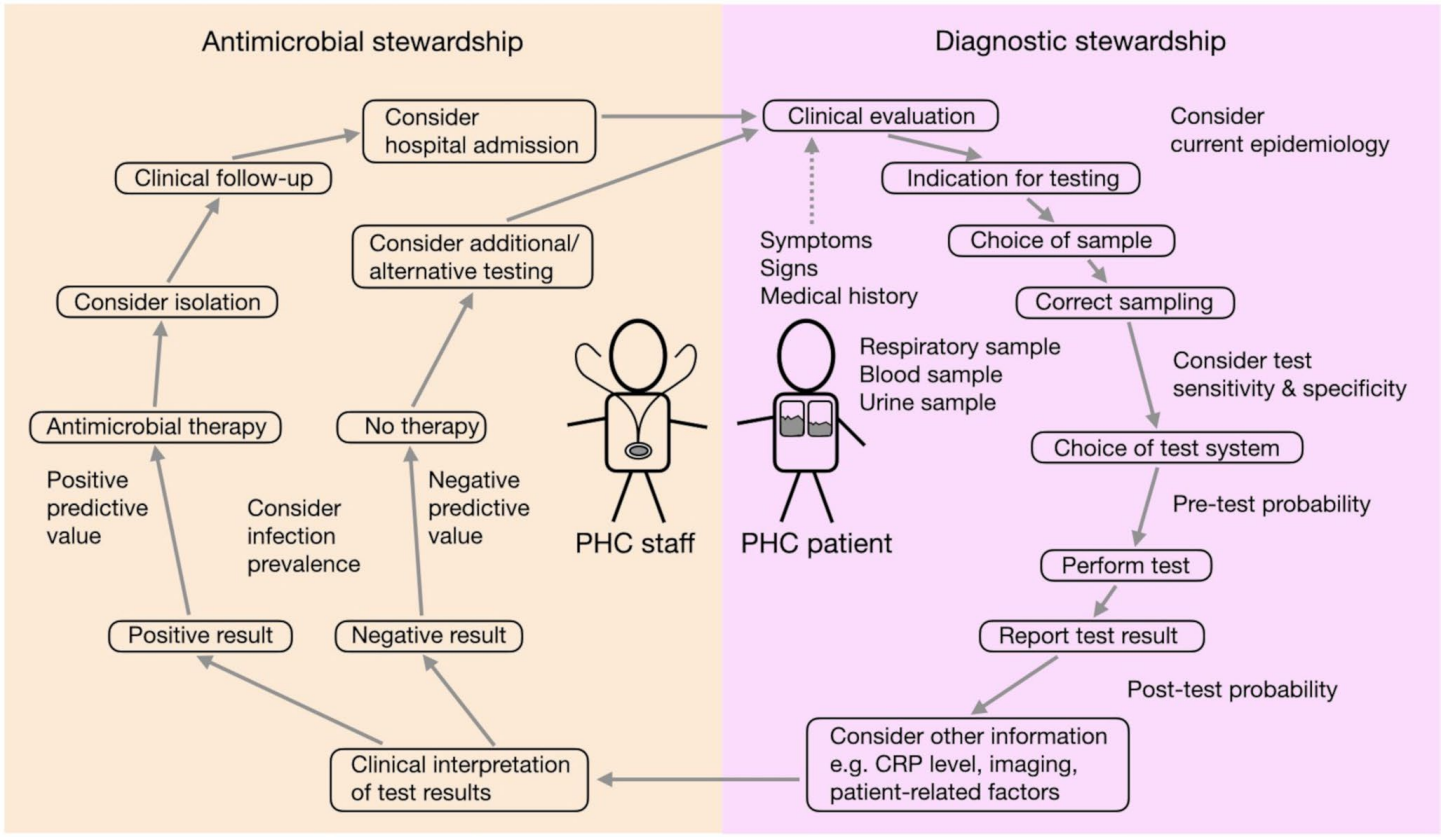
Selected considerations for diagnostic work-up and therapeutic intervention in PHC patients with RTI The process is initiated when a patient displaying symptoms and sings of a RTI seeks medical attention in a PHC center. Health care professionals make the indication for testing, collect the appropriate patient sample, choose the test system and perform the test. The careful interpretation of the test result in a clinical context is of paramount importance for optimal subsequent patient management. All these aspects and process steps can be incorporated in diagnostic and antimicrobial stewardship programs

RADTs are relatively simple to perform and typically do not require extensive laboratory infrastructure or expertise [52]. Therefore, RADTs are ideal for decentralized POCT, such as in doctor’s practices and PHC centers [29, 49, 53]. In PHC patients, whose clinical condition prior to testing may require subsequent admission to the hospital, an additional respiratory sample may be sent to the laboratory for PCR or culture due to their higher sensitivity to establish a definitive diagnosis and initiate antibiotic sensitivity testing [54–56]. In all other scenarios, the rapid turnaround time of RADTs can lead to immediate initiation of a specific antiviral or antibiotic therapy, thereby reducing the need for further, often unnecessary diagnostic investigations or empiric therapies [57]. However, due to their limited sensitivity, the World Health Organization (WHO) and European Centers for Disease Prevention and Control (ECDC) recommend to apply only RADTs of high diagnostic accuracy (≥80% sensitivity and ≥97% specificity as detailed below in the section ‘Considerations for practical use in PHC’) in symptomatic individuals or in high-prevalence settings such as influenza epidemics where rapid treatment decisions and isolation are critical [58, 59]. The clinical suspicion of a specific infection should always guide the diagnostic test of choice (Fig. 1).

### When to prefer nucleic acid testing?

Nowadays, both Reverse Transcription (RT)-PCR and LAMP tests are used for detecting respiratory pathogens, but RT-PCR systems may be more wide-spread in PHC. Both RT-PCR and LAMP tests are based on the specific amplification of microbial nucleic acids and display high sensitivity and specificity and short turn-around times [60]. There are several types of RT-PCR platforms including systems for POCT which allow for testing of respiratory samples in a unit-use cartridge or tube for specific pathogens such as influenza virus, RSV and SARS-CoV-2 [61–69] (Table 1). These systems have been used in PHC settings [70–73], and they are especially useful in situations where the highest accuracy is required, such as in severely immunocompromised patients in whom multiple different microbes can cause lower respiratory tract infection (LRTI) and where immediate initiation of treatment is desirable [72, 74–76]. Multiplex PCR systems may help to avoid unnecessary antibiotic therapy in viral infection, to more appropriate use of antiviral therapy and to better pathogen-directed antibiotic therapy, e.g. in infections with atypical pathogens or in the presence of resistance mechanisms [77–81]. In accordance with a statement by several medical societies, the routine use of multiplex PCR testing for pathogen panels is not recommended in community-acquired RTIs as long as clinical benefits for patient outcomes such as more targeted therapy with fewer adverse reactions, lower rates of hospitalization and shorter length of hospital stay have not been convincingly documented [82–86]. Rather, factors such as clinical symptoms and signs, medical and travel history, current epidemiology as well as other test results such as C-reactive protein (CRP) levels leading to a differential diagnosis should guide the rational choice of an efficient test systems while avoiding uneconomic or unnecessary tests [39, 41, 87]. Thus far, there is little evidence that the use of multiplex PCR in PHC will lead to improved patient outcomes or to more cost-effective delivery of care. This may go along with limited reimbursement of POCT to PHC providers.

However, multiplex PCR systems may be of advantage to differentiate between respiratory viruses when several of them are circulating in the community, to initiate targeted antibiotic therapy in otherwise undetected *B. pertussis*, *Chlamydia*, *Legionella* or *Mycoplasma* infections, in patients with severe RTI, in hospital and in ICU settings [37, 79, 81, 86, 88–90].

Based on the existing evidence, we recommend to consider multiplex PCR in PHC when the test result is likely to have a direct effect on the choice of antimicrobial therapy or on patient management e.g. when there is a high pre-test probability of an atypical pathogen or of antibiotic resistance.

### RADTs vs. PCR– pros and cons

RADTs and PCR tests each have distinct roles in the diagnosis of infections. RADTs provide quick, cost-effective solutions for immediate decision-making [91], while PCR tests offer the highest accuracy as required for vulnerable patients and in hospital settings [92, 93]. Comprehensive information on the diagnostic performance and availability of the test systems in use as well as on the current local epidemiology is required to choose the optimal test modality [94], highlighting the importance of diagnostic stewardship efforts (Fig. 1). The diagnostic accuracy of different RADTs is highly variable between manufacturers and products as well as between published studies [46, 50]. Some commercially available RADTs display huge differences between the sensitivities declared by the manufacturers in the instructions for use (IFU) and the clinical sensitivity in daily practice, making careful verification prior to implementation paramount for ID diagnostics [95–98].

One of the hallmarks of PCR is its high sensitivity, which results in the detection of minimum amounts of microbial nucleic acids. It is important to note and is often dismissed, that detection of microbial nucleic acids is not congruent with the diagnosis of an ongoing infection or its transmissibility [99, 100]. PCR results can remain positive for weeks and months after clearance of the infection, as we have learned from SARS-CoV-2 testing, or may be positive if the infection has been locally cleared by the immune system after pathogen exposure (Table 1) [101]. However, in such a scenario, cycle threshold (Ct) values are expected to be high. Therefore, pre-test evaluation of the patients and the critical estimation of the likelihood of a specific infection is central to the choice of a specific test (Fig. 1).

### Considerations for practical use in PHC

Several pre-analytical, analytical and post-analytical aspects are important for accurate microbiologic testing (Table 2). It is important that microbiologic testing is indicated and interpreted by healthcare professionals taking into consideration relevant factors such as clinical syndromes and pre-test probabilities, sensitivities and specificities of test systems in use, positive and negative predictive values, results of other diagnostic modalities as well as therapeutic consequences [102, 103] (Fig. 1). Additionally, reported symptoms and clinical signs are relevant to indicate appropriate sampling and diagnostic work-up e.g. for laboratory parameters such as CRP level, and imaging studies such as lung ultrasound or chest X-ray [104]. A CRP threshold of > 5 mg/dL offers a sensitivity of 75% and a specificity of 75% for the classification of LRTIs as of bacterial etiology [105]. Because CRP is transcriptionally induced in the liver upon cytokine stimulation, test results may be false negative for indication of a bacterial infection at early stages of disease onset (< 24 h) [106, 107]. However, in patients being symptomatic for more than 24 h, CRP detection offers a good negative predictive value to rule out bacterial infection [106, 107]. Lung ultrasound provides a sensitivity of 92% and a specificity of 90% for detecting pulmonary infiltrates at bed-site in bacterial CAP [105].

**Table 2:** Considerations for practical use of ID test systems in PHC

Category	Topic	Practical recommendations
Pre-analytical	Indications for microbiologic testing	Microbiologic testing should be integrated into diagnostic algorithms and stewardship programs [37, 112].
	Clinical syndrome	Consider the reported symptoms and clinical signs to indicate appropriate microbiological sampling and diagnostic work-up [239].
	Epidemiologic situation	Consider the pathogen activity in the community and possible exposure to assess pre-test probability of a specific infection [240].
	Travel history	Consider the travel history and possible exposure to assess pre-test probability of a specific infection [241].
	Type of specimen collected	Approved clinical specimens vary by test system. Consult the manufacturer’s instructions for use (IFU) for each test’s approved specimens. Combining specimens, such as nasal and oropharyngeal swabs, may increase sensitivity for respiratory viruses– if approved. Note that nasopharyngeal swabs are ideal for the detection of *B. pertussis* by PCR and that sputum samples (not nasopharyngeal swabs) are required for the diagnosis of infections with *S. pneumoniae*, *L. pneumophila* and *M. pneumoniae* by PCR [242, 243]. Specimens initially taken for RADTs may not be suitable for RT-PCR, and samples for PCR may not be suitable for subsequent culture.
	Timing of specimen collection	Respiratory samples for RADTs, PCR and culture are best taken in the first 1 to 3 days after symptom onset [48]. Urinary samples for UATs are also best taken in the first days after symptom onset, yet may contain a pathogen’s antigens for weeks [244, 245]. Expect serum samples for the diagnosis of *M. pneumoniae* or *B. pertussis* infections to be negative for pathogen-specific antibodies in previously unexposed/unvaccinated individuals in the first 7 to 10 days of the disease. Serial serum sampling may thus be required [39].
Analytical	In Vitro Diagnostic Regulations (IVDR)	Use IVDR-compliant or CLIA-waved tests.
	IFU	Follow the manufacturer’s IFUs carefully and ensure that the personnel performing a diagnostic test is documented to be trained and competent for the procedure.
	Overall diagnostic accuracy	Healthcare providers ought to verify that their chosen test system meets the requirements of diagnostic accuracy e.g. by comparing it to the gold standard in collaboration with their ID laboratory or microbiologic/virologic institute [111, 246].
	Sensitivity	RADTs for clinical use should ideally have a sensitivity of at least 80% aligning with recommendations from the ECDC, FDA, and WHO [109, 110].
	Specificity	RADTs for clinical use should ideally have a specificity of at least 97%, aligning with recommendations from the ECDC, FDA and WHO [109, 110].
Post-analytical	Clinical information	The exchange of clinical information including the patient’s history of contacts, travels and anti-infective therapy between physicians and the ID/microbiological laboratory is key for a correct interpretation of test results [112, 247].
	Other laboratory results	Consider other laboratory parameters such as CRP. A CRP threshold of > 5 mg/dL offers a sensitivity of 75% and a specificity of 75% for the classification of LRTIs as of bacterial etiology [105]. CRP is transcriptionally induced in the liver upon cytokine stimulation. CRP levels may thus be within the reference range (i.e. false negative for indication of a bacterial infection) at early stages of disease onset (< 24 h). However, in patients being symptomatic for > 24 h, normal CRP levels offer a good negative predictive value to rule out bacterial infection [107].
	Imaging studies	Consider the results imaging studies such as chest X-ray and lung ultrasound [104].
	Therapeutic consequences	Microbiologic testing should be integrated into therapeutic algorithms and stewardship programs ensuring that testing focuses on clinical cases in which test results are likely to have therapeutic consequences [37, 90, 164].

Approved clinical specimens vary by test system. Therefore, it is important to consult the manufacturer’s IFU for each test’s approved specimens. Combining specimens, such as nasal and oropharyngeal swabs, may increase sensitivity [108]. However, specimens initially taken for RADTs may not be suitable for RT-PCR, and samples for PCR may not be suitable for subsequent culture [48].

RADTs for clinical use should ideally have a sensitivity of at least 80% and a specificity of at least 97%, aligning with recommendations from the European ECDC, the US Food and Drug Administration (FDA), and the WHO [109, 110]. Healthcare providers are strongly encouraged to verify that their chosen test system meets these standards compared to PCR, in collaboration with their ID laboratory or microbiologic/virologic institute [111]. Useful information may come from randomized controlled trials (RCTs) evaluating these test systems. The exchange of clinical information including the patient’s history of contacts, travels and anti-infective therapy between physicians and the ID/microbiological laboratory is key for a correct choice and interpretation of test results [112].

In addition to the high diagnostic accuracy, ease of use, fast test results, economic efficiency and appropriate shelf-life are important to GPs when using POCT [113]. Furthermore, immediate decisions on patient management, the certainty of diagnostic and therapeutic decisions and improved communication with patients and practice processes are relevant reasons for GPs to perform POCT [113]. In contrast, high costs and lacking or inappropriate reimbursement hamper the use of POCT in PHC [114].

### Regulatory issues of testing in PHC

In general, test systems can be classified according to their technical complexity and the competence that health care professionals need to operate them [115]. For example, RADTs display low complexity and require low competence for use. PCR systems with unit-use cartridges have medium complexity and still require low competence for use, whereas automated PCR systems have medium complexity yet require high operator competence as obtained in the education of biomedical technicians. Therefore, automated PCR systems are typically performed in a laboratory environment.

In the EU, systems that only require low operator competence to deliver reproducible test results are regulated by the CE-IVDR (for In Vitro Diagnostic Regulation) for near patient testing (NPT) [115]. These test systems are intended for the use by health professionals outside a laboratory environment such as in PHC. Manufacturers must ensure that their test system is conform to the CE-IVDR, and Notified Bodies assess the validity of the manufacturer’s documents and issue a CE-certificate. In the US, the FDA has approved a range of tests for respiratory pathogens that are simple to perform and carry low risk of incorrect results as Clinical Laboratory Improvement Amendments (CLIA)-waived [116–118]. PHC providers that have obtained a CLIA certificate of waver can perform these tests at the POC. In countries outside the EU and the US, other regulations are relevant [119].

In conclusion, CE-certified or CLIA-waved tests can be employed in PHC settings by trained health care professionals. In addition, internal and external quality controls such as round robin tests are important to ensure their analytic performance [120–122].

### Considerations for specific pathogens

#### Influenza virus

The utility of clinical and epidemiological criteria to make the diagnosis of influenza has decreased since the emergence of SARS-CoV-2 in winter 2019/2020 [123]. Therefore, it is important to test symptomatic patients for possible influenza early after onset of the disease and independent of their vaccination history when there is moderate to high epidemiological influenza activity in the community or a precedent travel destination [124]. It is mainly children who have just received the nasal live influenza vaccine who should not be tested for several days [125]. The test system used should be able to detect both influenza A and B virus, and there are two main reasons for testing [126, 127]. First, to initiate antiviral treatment, when indicated, as early as possible but within 48 h after symptom onset and second, to avoid therapy with antibiotics in the absence of bacterial LRTI [128, 129].

In a systematic review, RADTs for the influenza virus showed a pooled sensitivity of 69% and a pooled specificity of 97% (summarized in Table 3). Only three RADTs used in over 100 studies reviewed had sensitivities ≥ 80% [105]. Subgroup analysis suggested better performance in children compared to adults [130]. Other studies, as well as our experience, indicate that the sensitivity of some RADTs for influenza virus ranges from 25 to 50% in daily practice [95, 131]. Therefore, a negative RADT result with a high clinical likelihood of influenza should be verified by a PCR test, which has higher sensitivity (see Table 3). However, due to its high specificity, a positive RADT result confirms the infection whereas a negative result with a high clinical likelihood of influenza should be verified by a PCR test [132]. PCR for influenza virus shows a pooled sensitivity of 94% and a pooled specificity of 98% [105]. Other nucleic acid amplification tests such as LAMP perform equally well or even better [133, 134]. Depending on the epidemiological context, multiplex PCR POCT systems detecting influenza virus A and B, RSV, and SARS-CoV-2 may be suitable for identifying these viral respiratory infections to initiate appropriate antiviral treatment and discontinue antibiotics [135, 136].

**Table 3:** Selected characteristics of selected test systems for specific respiratory pathogens

Pathogen	Antigenic tests	PCR	Serologic test	Comments
Influenza virus	sens. 69%, spec. 97% [105].	sens. 94%, spec. 98% [105].	NR [129].	Antigenic tests of moderate sensitivity may be sufficient to test symptomatic patients during influenza saison [248].
RSV	sens. 81–83%, spec. 97% [105, 137].	sens. 94%, spec. 97% [105].	NR [249].	Antigenic tests have substantially lower sensitivity in adults [137–139].
SARS-CoV-2	sens. 71–82%, spec. 99% [143–147].	sens. 95%, spec. 99% [143–147].	NR [250].	Antigenic tests have reduced sensitivity in children and for the Omicron variant [148, 150].
*S. pneumoniae*	sens. 66–81%, spec. 90–98% [159–161].	sens. 90–97%, spec. 61–96% [159–161].	NR [251].	UATs and conventional PCR from sputum cannot distinguish infection from asymptomatic carriage [177].
*L*. *pneumophila*	sens. 75–79%, spec. 99–100% [178, 179].	sens. 83–97%, spec. 90–98% [178, 179].	NR [252, 253].	UATs have limited ability to detect serogroups of *L. pneumophila* other than serogroup 1 or other *Legionella* species such as *L. bozemanii*, *L. longbeachae* and *L. micdadei*, which can still cause Legionnaires’ disease [187].
*M. pneumoniae*	NA.	sens. up to 97%, spec. up to 99% [105].	NR first-line [254]. sens. 85–90%, spec. 87–99% [105].	Molecular testing from sputum samples has the highest diagnostic accuracy [203]. PCR sensitivity is only around 38% in nasopharyngeal swabs [206]. If molecular testing is not available or comes back negative, sending serology for *Mycoplasma*-specific IgM and IgA to the lab may be considered [254, 255].
*B. pertussis*	NA.	sens. 90–94%, spec. 97–100% [212, 213].	NR first-line [256]. sens. 82%, spec. 82% [212, 213].	Serology is of limited use in PHC settings [257, 258]. Serology for pertussis toxin-specific IgG and IgA is suitable as case confirmatory test > 2 weeks after cough onset [216].

Sens.: sensitivity; spec.: specificity. NA: currently not available in Europe and the Americas. NR: not recommended for clinical decision-making

#### Respiratory syncytial virus (RSV)

In children, selected RADTs for RSV demonstrated pooled sensitivities ranging from 81 to 83% and a pooled specificity of approximately 97% [105, 137], suggesting a good clinical suitability for diagnosis in pediatric patients. However, in adults, RADTs for RSV displayed poorer sensitivities between 29% and 64% [137–139]. This highlights the importance of PCR testing in the elderly when there is a moderate to high clinical likelihood of viral bronchitis and RSV activity in the community. For PCR has a substantially higher sensitivity (Table 3), establishing it as today’s gold standard for diagnosing RSV infections, and making it the diagnostic modality of choice for residents of nursing homes and PHC patients who may require admission to the hospital [139]. Furthermore, some studies reported sensitivities of distinct test systems as low as 7.6% for RADTs in infants and 27% in adults [140, 141], rendering these test systems essentially ineffective for diagnostic purposes. Studies on over 22,000 respiratory samples show that viral loads for influenza A and B, RSV, SARS-CoV-2, and other viruses are similar [142], suggesting that the low sensitivities of some RADTs are largely analytical and attributable to the manufacturer’s test design.

Similar to the influenza virus, PCR for RSV shows a pooled sensitivity of 94% and a specificity of 97% [105], establishing it as today’s gold standard for diagnosing RSV infections in both children and adults, and making it the diagnostic modality of choice for residents of nursing homes and PHC patients who may require admission to the hospital.

### SARS-CoV-2

Several systematic reviews and meta-analyses have focused on RADTs for SARS-CoV-2. These studies reported pooled sensitivities that range from 71 to 82% and pooled specificities of around 99% [143–147]. Additionally, a systematic review and meta-analysis examined the diagnostic performance of SARS-CoV-2 RADTs in children, finding a moderate pooled sensitivity of 65.9% and specificity of 99.9% [148]. The sensitivity for SARS-CoV-2 is higher in symptomatic patients, when samples are taken within the first days of symptom onset and when the presumptive viral load is higher [46, 48, 144, 149]. Notably, RADT sensitivity was higher when using nasal or combined samples (e.g. combinations of nose, throat, mouth, or saliva samples) compared to nasopharyngeal samples [143]. In contrast, RADTs for SARS-CoV-2 showed reduced sensitivity for the Omicron variant, especially in samples with low viral loads [150]. It is worth mentioning that nasal sampling for RADTs does not require much specialized training. A study found similar accuracy between self-collected samples and those collected by healthcare personnel. Specifically, the sensitivity was 79% for self-collected samples and 83% for healthcare worker-collected samples, with specificities of 98% and 99%, respectively [151].

Overall, most RADTs are suitable for diagnosing COVID-19 in symptomatic PHC patients during the first days of symptom onset, especially in adults [152]. Yet, an additional respiratory sample may be sent to the laboratory for PCR in symptomatic patients who are likely to be hospitalized, when specific variants like Omicron are predominant and antigen test results remain negative despite high clinical suspicion. Similarly, with high clinical suspicion, repeated sampling and PCR testing for SARS-CoV-2 may be considered in immunocompromised patients [153].

Multiplex PCR systems for several respiratory viruses, such as influenza A and B, RSV, and SARS-CoV-2, are valuable for the early diagnosis of symptomatic patients [154]. Therefore, sending a respiratory sample to the laboratory for a multiplex PCR test may be indicated during winter time in the Northern Hemisphere when several epidemics with comparable clinical symptoms are evident within the same period [155]. Nonetheless, it should also be noted that multiplex PCRs can have lower sensitivities than single tests for a specific virus because of differences in the test design e.g. in primers or fluorophores [139, 156]. Similarly, multiplex antigen tests for detection of respiratory viruses may display lower sensitivity than single RADTs [157]. Once they have a considerably good sensitivity, multiplex antigen tests could serve as a cost-effective and easy-to-use diagnostic alternative to PCRs in symptomatic patients [158].

#### Streptococcus pneumoniae

There are UATs available for the detection of two clinically relevant bacteria causing CAP, i.e. *S. pneumoniae* and *L. pneumophila*. These UATs are suitable for POCT in doctor’s practices and PHC centers.

Systematic reviews and meta-analyses assessing the diagnostic accuracy of pneumococcal UATs report a pooled sensitivity of 66 to 81% and a pooled specificity of 90 to 98% in patients with pneumonia [159–161] (see Table 3). Even with this moderate sensitivity, UATs for *S. pneumoniae* increase CAP etiologic diagnosis by about 20%.

The early identification of *S. pneumoniae* guides antibiotic therapy and allows for the safe de-escalation with no relevant risk of clinical failure [162, 163].

Furthermore, de-escalation of therapy to penicillins, aminopenicillins or ceftriaxone reduces drug toxicities and counter-acts the emergence of antimicrobial resistance [164]. However, evidence suggests that in daily practice, a positive UAT does not greatly change patient management, the costs for therapy or the length of hospital stay in patients requiring hospital admission [165].

False-positive results from pneumococcal UAT may occur in patients with a CAP episode within the previous 3 months, in patients with chronic respiratory diseases colonized with *S. pneumoniae* and within the first days after pneumococcal vaccination [162]. Furthermore, there is evidence that sensitivities of UATs have decreased from 2011 to 2015 [166], possibly associated with the introduction of the 13-valent pneumococcal polysaccharide vaccine [167]. Therefore, ongoing studies will be required to monitor the diagnostic accuracy of the UATs and re-assess their clinical utility and their sensitivity with newly emerging serovars which have replaced *S. pneumoniae* strains covered by the different vaccines [168].

It is not only in patients with negative UATs but also in those with severe LRTI who may need hospitalization [169], that a respiratory sample should be sent for culture or PCR to identify the pathogen and to assess antibiotic susceptibility of isolates which is specifically of importance in regions with high penicillin or macrolide resistance among pneumococci. Importantly, the sensitivity of sputum culture is limited due to multiple factors such as inadequate sample quality [170], long sample storage and transport or after the initiation of antimicrobial therapy [171]. Using culture as reference method, PCR from sputum samples substantially increases the rate of detection of bacteria [172]. Several studies on pneumococcal CAP demonstrate that PCR from sputum provides a high sensitivity and specificity [173–176] (see Table 3). However, as the sensitivity of the PCR test in use increases, its specificity decreases because more colonizations with *S. pneumoniae* are mis-classified as infections [177]. This highlights the importance of diagnostic stewardship programs which help selecting clinical cases for the appropriate microbiologic test.

#### Legionella pneumophila

Infections with *L. pneumophila* account for less than 3% of CAP cases and are commonly diagnosed with UATs, which only detect serogroup 1 [13]. Systematic reviews and meta-analyses indicate that UATs for *L. pneumophila* have pooled sensitivities between 75% and 79%, and pooled specificities between 99% and 100% [178, 179]. The importance of rapid diagnostic methods, such as UATs, is highlighted by the association of delayed therapy with poorer clinical outcomes in *Legionella* infections [180, 181]. A positive *Legionella* UAT should prompt immediate adjustment of antibiotic treatment to a fluoroquinolone (levofloxacin or moxifloxacin) or an intravenous macrolide (preferably azithromycin) [182, 183].

*Legionella* antigens can be detected in urine as early as 1 to 3 days after symptom onset and may remain detectable for weeks [184]. Generally, the antigen is no longer present 1 to 2 months after treatment [185, 186]. However, UATs have limited ability to detect serogroups of *L. pneumophila* other than serogroup 1 or other *Legionella* species, especially in regions where they are endemic and in immunocompromised patients [187]. PHC patients with a negative UAT but a high clinical suspicion of *Legionella* pneumonia or severe LRTI should thus be referred to the hospital for further diagnostic work-up and treatment. Particularly, moderate to high CRP levels, low serum sodium and phosphate concentrations, and high creatine kinase levels, should prompt taking a respiratory sample for PCR or culture. Interestingly, a CRP level below 13 mg/dL can rule out *L. pneumophila* serogroup 1 infections in immunocompetent patients with symptoms lasting at least 48 h [188].

Most current test systems do not detect non-*pneumophila Legionella* species, but alternative systems are being developed. A multiplex RT-PCR that detects all *Legionella* species and identifies *L. pneumophila* simultaneously in respiratory samples would be a significant advancement for modern microbiological laboratories specifically when taking care of immunocompromised patients and when other tests remain inconclusive in spite of high clinical suspicion [189, 190]. Despite the high specificity of UATs, false positive results can occur, such as in cases of *Pseudomonas* infection or colonization [191].

All *Legionella* species can be cultured, which remains the gold standard for diagnosing legionellosis. However, cultures are limited by the slow growth of *Legionella*, often requiring 5 days or more, and by the fact that only half of Legionnaires’ disease patients produce sputum. RT-PCR can detect *Legionella* in respiratory specimens, urine, and blood [192, 193]. PCR offers numerous advantages, including higher sensitivity compared to culture and rapid results, typically available within 4 h [194, 195]. Meta-analyses report pooled sensitivities of 83 to 97% and specificities of 90 to 98% [196, 197]. Compared to UATs, PCR in respiratory samples can diagnose 18 to 30% more legionellosis cases [196].

LAMP technology appears even more sensitive than PCR for detecting *Legionella* in respiratory and environmental samples [198, 199].

#### Mycoplasma pneumoniae

As of April 2025, there are no clinical studies on IVDR-compliant RADTs for *M. pneumoniae* available in Europe and the Americas. The Ribotest Mycoplasma^®^, a RADT available in Japan, has a sensitivity of 62.5 to 71% and specificity of 89 to 90.9% compared to PCR [200, 201]. The DK-MP-001 RADT shows 81.7% sensitivity and 100% specificity compared to LAMP [202].

*M. pneumoniae* infections are commonly detected by PCR from a respiratory sample in PHC settings [203], and different commercially available assays provide sensitivities between 83 and 97% [105, 204, 205] (see Table 3).

In settings, where molecular tests for *M. pneumoniae* are not available or not reimbursed, the serologic diagnosis of *M. pneumoniae* infections with enzyme-linked immunosorbent assay (ELISA) may still be in use. A systematic review of 7 studies found the pooled sensitivity and specificity for *M. pneumoniae* IgG and IgM to be 85% and 90%, respectively. This is close to PCR, which has an even higher sensitivity and specificity [105] (see Table 3), but requires shipment of a sputum sample to the ID laboratory. In contrast to sputum samples, nasopharyngeal swabs are not appropriate for the diagnosis of *M. pneumoniae* infections because their use for PCR results in a sensitivity of only around 38% [206, 207].

*M. pneumoniae* can be found in the respiratory tract of asymptomatic individuals. In healthy children for example, *M. pneumoniae* DNA could be detected in 21 to 56% of subjects tested for suspicion of infection with considerable geographic and seasonal variation [208, 209]. Importantly, neither serology nor PCR nor culture appear to enable clear differentiation between asymptomatic carriage and infection [208]. Therefore, it is important to test individuals with a moderate to high likelihood of actual *Mycoplasma* LRTI [210].

#### Bordetella pertussis

Currently, there are also no clinical studies on commercially available IVDR-compliant RADTs for *B. pertussis* for use in PHC settings. Consequently, the definitive diagnosis of whooping cough requires shipment of appropriate samples to the laboratory for PCR, culture, or serological tests (see Tables 1 and 3). An academic study developed an immunochromatographic RADT for *B. pertussis* for clinical use. This test demonstrated a sensitivity of 86.4% and a specificity of 97.1% in a prospective multicenter RCT involving 195 patients [211], suggesting that the technical challenges and precision requirements for a commercially available RADT can be met.

PCR is the most sensitive method for detecting *B. pertussis*, reaching a sensitivity of 90 to 94% and a specificity of 97 to 100% in prospective studies [212, 213]. The fast turnaround time and high throughput capability of PCR testing facilitate outbreak management. A notable phenomenon in pertussis diagnostics are pseudo-outbreaks, potentially due to the detection of *B. holmesii* or environmental contamination by intact or residual bacteria or vaccine components [214]. This has implications for clinical sample collection, laboratory processing, and test selection. PCR targeting multiple regions is less likely to detect non-pertussis *Bordetella* species, and culture is recommended in potential outbreak investigations.

PCR from a nasopharyngeal swab appears to be the optimal method for diagnosing whooping cough within the first 2 weeks of symptom onset [215]. In contrast, ELISA for *B. pertussis*-specific IgG and IgA is unsuitable for timely diagnosis in the early stages because antibodies take about 2 weeks to rise and 2 to 6 months to peak [212, 216, 217]. Serology however, e.g. for pertussis toxin-specific IgG and IgA, may identify additional clinical cases of whopping cough in adolescents and adults who seek treatment more than 2 weeks after cough onset, i.e. after the optimal interval for nasopharyngeal sampling, or after culture results have returned negative, provided that they have not been recently vaccinated [212]. The latter is attributable to the fact that the interpretation of ELISA results is complicated by humoral responses post-vaccination, by maternal antibodies in infants as well as in preschool children who have a limited IgA response to specific *Bordetella* antigens [217, 218]. Consequently, two serum samples, taken 2 weeks apart, may be needed for a retrospective serological diagnosis, delaying the optimal time window for antibiotic therapy initiation and infection chain interruption. Thus, in such inconclusive diagnostic cases, decision on therapy has to be based on epidemiological and more importantly on clinical evaluation of the specific patient case.

## Limitations

Worldwide, PHC is provided in a variety of settings. Most of the original and secondary evidence summarized in this review are from Western countries. Therefore, it is difficult to derive definitive recommendations for low-and-middle-income countries. Even in Western countries, the use of POCT systems varies considerably between different PHC providers. For example, in most countries POCT systems are not commonly used by community nurses or pharmacies [219, 220]. Also, different logistic and reimbursement strategies affect the availability and frequency of testing for respiratory pathogens [113]. As a consequence, guidelines and recommendations for the use of POCT in PHC should be validated for each disease entity and each specific setting as demonstrated for POCT for infections with group A beta-haemolytic *Streptococcus* [221].

Some of the evidence discussed such as data on the molecular detection of resistance genes are from hospitalized patients and cannot be directly be extrapolated to PHC settings. Furthermore, data is scarce on how testing for respiratory pathogens can improve not only antimicrobial therapy but clinical outcomes of RTIs.

## Conclusions

Clinically useful RADT and PCR test systems enable the etiological diagnosis of respiratory tract infections in PHC [70, 222, 223]. Yet, evidence suggests that not all test results significantly influence patient management or reduce the inappropriate use of antibiotics in viral RTIs [131, 135, 224, 225]. For instance, a clinical study conducted from 2002 to 2004 found that detecting a respiratory virus using a multiplex PCR panel led to cessation of antibiotic therapy in a minority of cases, with ß-lactam antibiotics often being continued [226]. However, more recent studies provide a different perspective. From 2012 to 2014, a study of 323 patients with CAP found that multiplex RT-PCR for respiratory pathogens identified causative agents in 87% of patients compared to a 39% detection rate with culture-based methods. RT-PCR results enabled antibiotic therapy de-escalation in 77% of patients [172]. Vice versa, upon detection of influenza virus, a specific anti-viral therapy could be initiated in that study and in another one from 2017 [73, 172]. PCR tests positive for bacterial RTI led to antibiotic therapy initiation or adaptation in 19% of pneumonia episodes, while negative PCR results led to non-initiation or discontinuation of antibiotics in 28% [227]. Avoiding the over-prescription of antibiotics reduces adverse events and antimicrobial resistance and subsequent expenses [228–230]. Therefore, POCT for the clear identification of respiratory pathogens can be cost-effective in PHC [61, 229, 231]. Furthermore, diagnostic and antimicrobial stewardship efforts and careful interpretation of results in a clinical context are of paramount importance (Fig. 1). Incorporating modern diagnostic tools into protocols at PHC facilities has the potential to improve patient care and flow management, optimize resource allocation, and enhance ID control [232]. We hope that continued investment in diagnostic technologies and integrated approaches and its linkage to clinical expertise and proper test selection will further improve the specific diagnosis of RTIs, and subsequently the quality of patient management and epidemiological surveillance.

## Data Availability

No datasets were generated or analysed during the current study.

## Abbreviations

*B. pertussis*: *Bordetella pertussis*
CAP: Community-acquired Pneumonia
CLIA: Clinical Laboratory Improvement Amendments
COVID: Coronavirus Disease
CRP: C-reactive Protein
ECDC: European Centers for Disease Prevention and Control
ELISA: Enzyme-linked immunosorbent assay
GP: General Practitioner
ID: Infectious Disease
IFU: Instructions for Use
IVDR: In Vitro Diagnostic Regulation
*L. pneumophila*: *Legionella pneumophila*
LRTI: Lower Respiratory Tract Infection
*M. pneumoniae*: *Mycoplasma pneumoniae*
NPT: Near Patient Testing
PCR: Polymerase Chain Reaction
PHC: Primary Health Care
POCT: Point of care Testing
RADT: Rapid Antigen Detection Test
RSV: Respiratory Syncytial Virus
RTI: Respiratory Tract Infection
RT: Reverse transcription
SARS-CoV-2: Severe Acute Respiratory Syndrome Coronavirus Type 2
*S. pneumoniae*: *Streptococcus pneumoniae*
UAT: Urinary Antigen Test
WHO: World Health Organization
